# Supplementary material for: Profile and clinical implication of circular RNAs in human papillary thyroid carcinoma
Source: PeerJ. 2018 Aug 7;6:e5363. doi: 10.7717/peerj.5363 (PMC6086080; doi:10.7717/peerj.5363)
Supplement: Supplemental Information 2 [file peerj-06-5363-s002.docx]

**Profile and clinical implication of circular RNAs in human papillary thyroid carcinoma**

Table S1. The primers for circRNAs and GAPDH.

| Primer names |  | Sequences (5’->3’) |
| --- | --- | --- |
| hsa_circRNA_047771-F |  | AAATGATCCAAGTCTCCCAG |
| hsa_circRNA_047771-R |  | AAAGCCTTTAGACCTGTTTT |
| hsa_circRNA_101408-F |  | ATTGCTACTGCCTGTAAAAC |
| hsa_circRNA_101408-R |  | GTTAATGTAACGGGAGACCA |
| hsa_circRNA_014213-F |  | TGCTCAGAATTCATCTGAAGA |
| hsa_circRNA_014213-R |  | GGAACTCATGCTGCAACAGT |
| hsa_circRNA_090446-F |  | TGTTCCCACTCCCATGCTTTCT |
| hsa_circRNA_090446-R |  | TTACTCATCCACCCACCATCA |
| hsa_circRNA_007148-F |  | TAGTGGTCCCGGAATTAAGA |
| hsa_circRNA_007148-R |  | AGTGTTCCATCCTCTGCTCT |
| hsa_circRNA_004662-F |  | GAGCACGCTTACTACCTTCA |
| hsa_circRNA_004662-R |  | CAAGCCATGTATCTTTCAGTT |
| hsa_circRNA_046843-F |  | CAGAGGAGTTGGAGTTGCTA |
| hsa_circRNA_046843-R |  | CTTGCTCACATCACTTCGTT |
| hsa_circRNA_061346-F |  | GCCGATGATGACGAGGACGAT |
| hsa_circRNA_061346-R |  | GCCGATGATGACGAGGACGAT |
| GAPDH-F |  | ACCCACTCCTCCACCTTTGAC |
| GAPDH-R |  | TGTTGCTGTAGCCAAATTCGTT |

Table S2. Association between three upregulated and three downregulated circRNAs expression levels and clinicopathological characteristics in 40 PTC patients

|  | hsa_circRNA_004662**^#^** | |  | hsa_circRNA_046843**^#^** | |  | hsa_circRNA_061346**^#^** | |  | hsa_circRNA_405498**^&^** | |  | hsa_circRNA_101408**^&^** | |  | hsa_circRNA_014213**^&^** | |
| --- | --- | --- | --- | --- | --- | --- | --- | --- | --- | --- | --- | --- | --- | --- | --- | --- | --- |
|  | High expression | Low expression |  | High expression | Low expression |  | High expression | Low expression |  | High expression | Low expression |  | High expression | Low expression |  | High expression | Low expression |
| Age(years) |  |  |  |  |  |  |  |  |  |  |  |  |  |  |  |  |  |
| ≥45 | 12(50) | 9(56.3) |  | 15(57.5) | 6(42.9) |  | 11(61.1) | 10(45.5) |  | 9(42.9) | 12(57.1) |  | 13(56.5) | 8(42.1) |  | 6(42.9) | 15(57.7) |
| <45 | 12(50) | 7(43.7) |  | 11(42.7) | 8(57.1) |  | 7(38.9) | 12(54.5) |  | 12(57.1) | 7(42.9) |  | 10(43.5) | 11(57.9) |  | 8(57.1) | 11(42.3) |
| Sex |  |  |  |  |  |  |  |  |  |  |  |  |  |  |  |  |  |
| Female | 15(78.9) | 15(71.4) |  | 18(81.8) | 12(66.7) |  | 14(82.4) | 16(69.6) |  | 18(85.7) | 12(63.2) |  | 13(68.4) | 17(77.3) |  | 11(73.3) | 19(76) |
| male | 4(21.1) | 6(28.6) |  | 4(18.2) | 6(33.3) |  | 3(17.6) | 7(30.4) |  | 3(14.3) | 7(36.8) |  | 6(31.6) | 5(22.7) |  | 4(26.7) | 6(24) |
| Hashimoto |  |  |  |  |  |  |  |  |  |  |  |  |  |  |  |  |  |
| Yes | 4(16.7) | 2(12.5) |  | 3(21.4) | 3(11.5) |  | 2(13.3) | 4(16) |  | 4(19.0) | 2(10.5) |  | 3(16.7) | 3(13.6) |  | 1(5.3) | 5(23.8) |
| No | 20(83.3) | 14(87.5) |  | 11(78.6) | 23(88.5) |  | 13(86.7) | 21(84) |  | 17(81) | 17(89.5) |  | 15(83.3) | 19(86.4) |  | 18(94.7) | 16(76.2) |
| TIRAID |  |  |  |  |  |  |  |  |  |  |  |  |  |  |  |  |  |
| 4a | 2(9.1) | 2(11.1) |  | 2(8.3) | 2(12.5) |  | 2(12.5) | 2(8.3) |  | 1(5.6) | 3(13.6) |  | 2(10) | 2(10) |  | 2(9.5) | 2(10.5) |
| 4b | 5(22.7) | 8(44.4) |  | 10(41.7) | 3(18.8) |  | 4(25) | 9(37.5) |  | 6(33.3) | 7(31.8) |  | 6(30) | 7(35) |  | 8(38.1) | 5(26.3) |
| 4c | 15(68.2) | 8(44.4) |  | 12(50) | 11(68.8) |  | 10(62.5) | 13(54.2) |  | 11(61.1) | 12(54.5) |  | 12(60) | 11(55) |  | 11(52.4) | 12(63.2) |
| BRAF mutation |  |  |  |  |  |  |  |  |  |  |  |  |  |  |  |  |  |
| Yes | 12(57.1) | 5(25)**^*^** |  | 10(43.5) | 6(35.3) |  | 9(47.4) | 7(33.3) |  | 5(25) | 11(55) |  | 6(27.8) | 10(45.5) |  | 7(26.9) | 9(64.3)**^*^** |
| No | 9(42.9) | 15(75) |  | 13(56.5) | 11(64.7) |  | 10(52.6) | 14(66.7) |  | 15(75) | 9(45) |  | 17(72.2) | 7(54.5) |  | 19(73.1) | 5(35.7) |
| Tumor size (cm) | 1.6(1.85-2.07) | 1.2(0.8-2.0) |  | 1.3(0.95-2.05) | 1.2(0.8-2.0) |  | 1.4(1.1-2.15) | 1.3(1.0-2.0) |  | 1.3(0.8-2.0) | 1.2(0.8-2.0) |  | 1.2(0.8-2.0) | 1.3(1-2.05) |  | 1.4(1.09-2.15) | 1.3(0.8-2.0) |
| Stage (AJCC) |  |  |  |  |  |  |  |  |  |  |  |  |  |  |  |  |  |
| I-II | 13(68.4) | 12(57.1) |  | 15(65.2) | 10(58.8) |  | 18(75) | 7(43.8)**^*^** |  | 15(71.4) | 10(52.6) |  | 16(80) | 9(45)**^*^** |  | 12(66.7) | 13(59.1) |
| III-V | 6(31.6) | 9(42.9) |  | 8(34.8) | 7(41.2) |  | 6(25) | 9(56.3) |  | 6(28.6) | 9(47.4) |  | 4(20) | 11(55) |  | 6(33.3) | 9(40.9) |
| Lymph node metastasis | |  |  |  |  |  |  |  |  |  |  |  |  |  |  |  |  |
| Yes | 12(63.2) | 6(28.6)**^*^** |  | 13(59.1) | 5(27.8)**^*^** |  | 11(52.4) | 7(36.8) |  | 5(26.3) | 13(61.9)**^*^** |  | 7(36.8) | 11(52.4) |  | 8(42.1) | 10(47.6) |
| No | 7(36.8) | 15(71.4) |  | 9(40.9) | 13(72.2) |  | 10(47.6) | 12(63.2) |  | 14(73.7) | 8(38.1) |  | 12(63.2) | 10(47.6) |  | 11(57.9) | 11(52.4) |
| Focality |  |  |  |  |  |  |  |  |  |  |  |  |  |  |  |  |  |
| Unifocal | 8(40) | 5(25) |  | 4(28.6) | 9(34.6) |  | 7(43.8) | 6(25) |  | 8(23.8) | 5(42.1) |  | 6(25) | 7(43.8) |  | 4(21.1) | 9(42.9) |
| Multifocal | 12(60) | 15(75) |  | 10(71.4) | 17(65.4 |  | 9(56.3) | 18(75) |  | 16(76.2) | 11(57.9) |  | 18(75) | 9(56.2) |  | 15(78.9) | 12(57.1) |

**^#^**The upregulate and **^&^**downrelated circRNAs. The mean expression value for circRNA was used as the cutoff scores for the high-expression and low-expression of circRNAs. Data are expressed as n (percent) or median (interquartile range); circRNA, circular RNA; PTC, papillary thyroid carcinoma; TIRAIDS, thyroid imaging reporting and data system; TNM, tumor-node-metastasis; LNM, lymph node metastasis.**^*^** *P* <0.05.


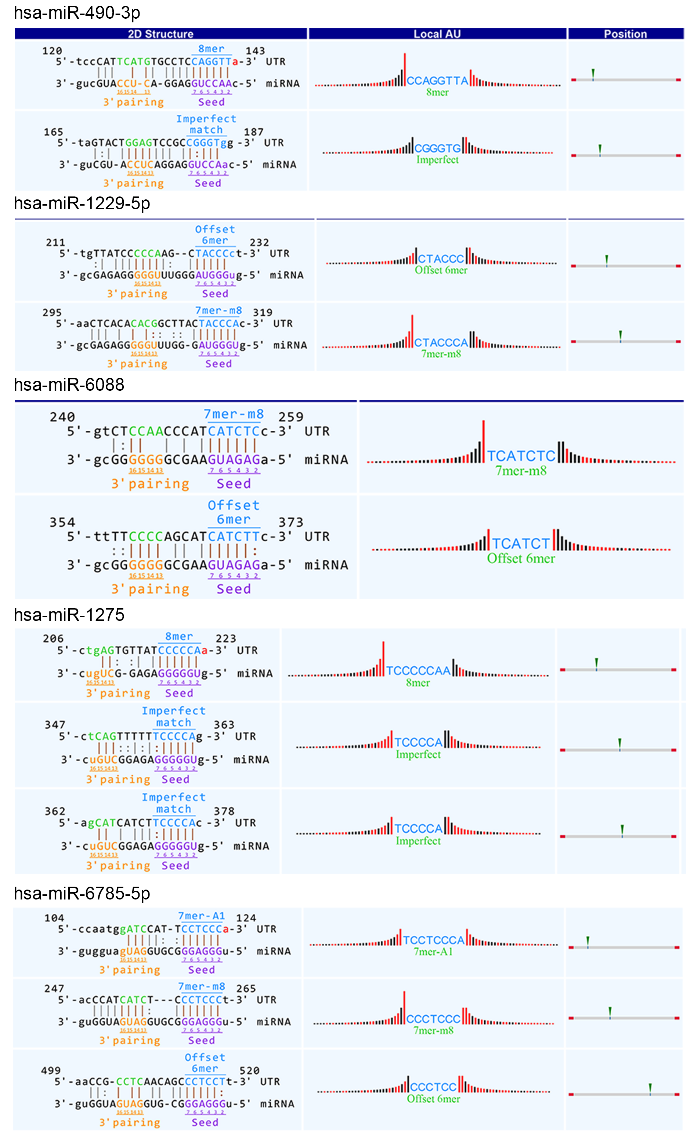


Fig S1. The top 5 ranking MREs targets for has_circRNA_007148. The 2D structure shows the MRE sequence, the target miRNA seed type (7mer-m8, 8mer, 6mer, offset 6mer, imperfect) and the pairing of target miRNA nucleotides 13-16. The precise base positions are displayed in the alignments in the upper left and right corners.


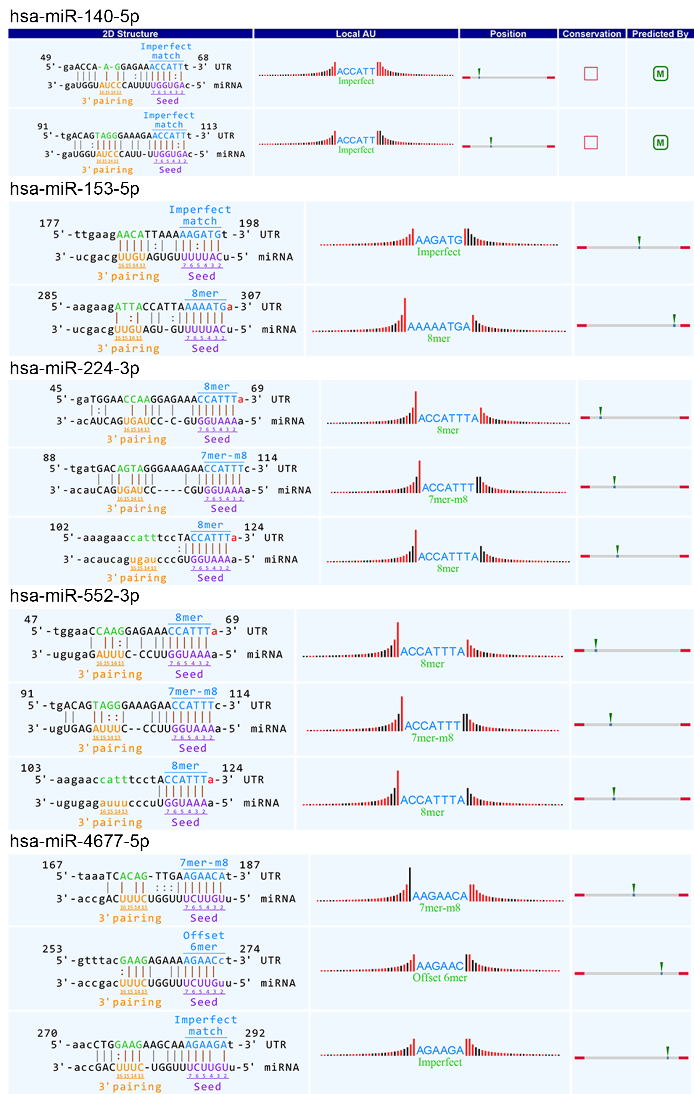


Fig S2. The top 5 ranking MREs targets for hsa_circRNA_0047771. The 2D structure shows the MRE sequence, the target miRNA seed type (7mer-m8, 8mer, 6mer, offset 6mer, imperfect) and the pairing of target miRNA nucleotides 13-16. The precise base positions are displayed in the alignments in the upper left and right corners.


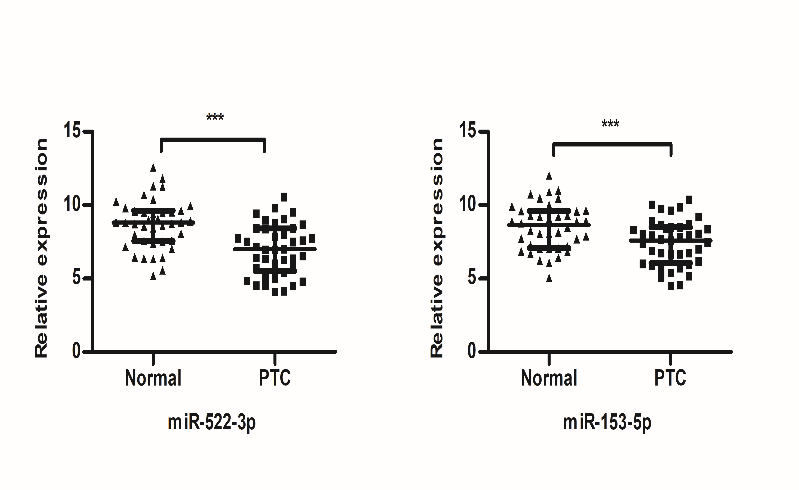


Fig S3. The relative expression of miR-522-3p and miR-153-5p were validated in 40 human PTC tissues and adjacent non-tumorous tissues. ^***^*P*<0.001. Normal: adjacent non-tumorous tissue; PTC: papillary thyroid tumor tissue. ΔCt values were used to assess the relative gene expression, which was normalized to U6 expression level.
